# Non-parenchymal cells: key targets for modulating chronic liver diseases

**DOI:** 10.3389/fimmu.2025.1576739

**Published:** 2025-06-11

**Authors:** Tongwang Yang, Zhiyun Gu, Juan Feng, Juanjuan Shan, Cheng Qian, Na Zhuang

**Affiliations:** Chongqing Key Laboratory of Translational Research for Cancer Metastasis and Individualized Treatment, Chongqing University Cancer Hospital, School of Medicine, Chongqing University, Chongqing, China

**Keywords:** chronic liver disease, non-parenchymal cells, innate immunity, therapeutic targeting, liver fibrosis

## Abstract

Non-neoplastic chronic liver diseases (CLDs), including alcoholic liver disease, metabolic-associated fatty liver disease, viral hepatitis, fibrosis, and cirrhosis, pose a global health challenge due to progressive fibro-inflammatory remodeling. Emerging evidence highlights the pivotal roles of non-parenchymal cells (NPCs)—liver sinusoidal endothelial cells (LSECs), hepatic stellate cells (HSCs), Kupffer cells (KCs), and innate immune lymphocytes such as natural killer (NK) and natural killer T (NKT) cells—in driving disease progression. Chronic liver injury triggers LSEC capillarization, HSC transdifferentiation into collagen-producing myofibroblasts, and KC polarization toward pro-inflammatory phenotypes, collectively exacerbating extracellular matrix deposition and immune dysregulation. Dysfunctional NK/NKT cells play dual roles in antiviral defense and fibrosis amplification through excessive cytokine production. This review summarizes recent advances in understanding NPC-driven mechanisms underlying chronic liver injury and fibrosis, with a focus on LSEC dysfunction, HSC activation, and inflammation mediated by KCs and NK/NKT cells. Furthermore, we delve into emerging therapeutic strategies aimed at targeting NPC-specific pathways, including mechanotransduction modulation in LSECs, metabolic reprogramming of HSCs, and regulation of KC polarization. These approaches provide valuable insights into halting CLD progression and advancing the development of innovative antifibrotic therapies.

## Introduction

Chronic liver diseases (CLDs), including alcoholic liver disease (ALD), metabolic-associated fatty liver disease (MAFLD), viral hepatitis, fibrosis and cirrhosis, are key drivers of liver cancer development and contribute significantly to the high global mortality associated with liver diseases (1–4). There are regional variations in the etiology of liver cancer: chronic hepatitis B virus (HBV) infection remains the leading cause of hepatocellular carcinoma (HCC) in Asia (5), while metabolic dysfunction-associated steatohepatitis (MASH) has become the predominant risk factor in Western populations (6). Other contributing factors, such as aflatoxin exposure, alcohol misuse, tobacco use, obesity, and diabetes, further accelerate the progression of CLDs (7–10). If left unmanaged, these underlying conditions often lead to cirrhosis, with approximately 85% of cirrhotic patients ultimately progressing to HCC (11). Therefore, gaining deeper insights into the pathogenesis of CLDs is crucial for the early prevention of HCC.

The liver is a vital immune organ enriched with diverse cell populations (12). It comprises 80% parenchymal cells (hepatocytes) and 20% non-parenchymal cells (NPCs) (13). NPCs include liver sinusoidal endothelial cells (LSECs), hepatic stellate cells (HSCs), Kupffer cells (KCs), and innate lymphocytes such as NK and NKT cells (12, 14). Together, parenchymal and non-parenchymal cells form the structural basis of hepatic lobules: hepatocytes are organized into cords, while NPCs reside in the sinusoidal compartment. Specifically, LSECs line the sinusoids, KCs (liver-resident macrophages) are mainly localized within sinusoidal lumens, and HSCs occupy the perisinusoidal space of Disse (15). Interactions between these cells and sinusoid-dwelling NK/NKT cells are crucial for hepatic immunity and disease pathogenesis.

The development of CLD involves multifaceted cellular dysfunction. For instance, LSEC capillarization impairs their physiological functions (16), while HSC activation drives their transformation into extracellular matrix (ECM)-producing myofibroblasts (17). Concurrently, KCs polarize into M1 phenotypes, releasing mediators such as tumor necrosis factor (TNF)-α, interleukin (IL)-1β, and IL-6 (18). NK and NKT cells further aggravate fibrosis and inflammation (19, 20). Among these, HSC activation is a pivotal event in CLD progression. Research indicates that activated HSCs release extracellular vesicles (EVs) during the early stages of activation, which induce pro-inflammatory KC polarization via Toll-like receptor 4 (TLR4) signaling (21). Interestingly, LSEC-derived EVs counteract fibrogenic phenotypes in activated HSCs and mitigate KC-mediated inflammation (22). Lipopolysaccharide (LPS) also activates LSECs to enhance IL-12/IL-18-driven interferon (IFN)-γ production in NK cells. IFN-γ promotes LSEC secretion of CXCL10, recruiting additional NK cells to amplify hepatic inflammation (16). Conversely, NK/NKT cell-derived IFN-γ can also attenuate liver injury (23). Modulating NK/NKT cell activity presents promising therapeutic opportunities. Activation of the E-prostanoid 3 receptor (EP3) enhances NK cell adhesion and cytotoxicity toward HSCs (24), while fasudil, a RhoA kinase inhibitor, attenuates fibrosis by activating NK cells and inhibiting HSC activation (25). These findings underscore the importance of understanding NPC interactions in CLD treatment.

This review highlights recent advances in elucidating the roles of liver-specific NPCs, including LSECs, HSCs, KCs, and innate lymphoid subsets (NK/NKT cells) in chronic liver injury, fibrosis, and cirrhosis. We emphasize their mechanistic contributions to CLD pathogenesis and explore emerging therapeutic strategies aimed at targeting these cells to reshape disease progression.

## Liver sinusoidal endothelial cells

LSECs are a unique and highly specialized endothelial cell population, representing the most abundant NPC type that lining the sinusoidal capillaries of the liver, characterized by their plentiful fenestrae, absence of a continuous basement membrane, and lack of diaphragms. LSECs establish a highly permeable barrier between the bloodstream and hepatic parenchyma, playing a pivotal role in maintaining liver homeostasis (26). Under normal physiological conditions, LSECs regulate hepatic vascular tone and maintain the quiescent state of hepatic stellate cells, thereby inhibiting intrahepatic vasoconstriction and the development of fibrosis (27, 28). However, under pathological conditions, LSECs undergo capillarization, a process that promotes angiogenesis, vasoconstriction, and the release of vascular signaling molecules that drive the progression of liver fibrosis (16). For instance, in liver fibrotic models induced by carbon tetrachloride or bile duct ligation, levels of adipocyte fatty acid binding protein (A-FABP) derived from LSECs are elevated. This elevation activates the Hedgehog signaling pathway, facilitating LSEC capillarization (29). Capillarized LSECs undergo partial endothelial-to-mesenchymal transition, acquiring a myofibroblast-like phenotype that further promotes ECM synthesis and results in the deposition of perisinusoidal ECM, thereby exacerbating liver fibrosis (30). Recent studies have indicated that employing agents to inhibit ECM deposition can decrease ECM stiffness, potentially reversing LSEC capillarization. This finding opens new avenues for the treatment of diseases associated with liver fibrosis (31).

In addition to capillarization, the functional impairment of LSECs induced by liver injury is also a critical factor in the early development of liver diseases such as fibrosis and hepatitis. Aging is considered to increase susceptibility to CLD and accelerate the progression of liver fibrosis. Dai et al. found that aging leads to LSEC dysfunction by downregulating the expression of SIRT1 in hepatocytes, which subsequently activates HSCs and exacerbates the deterioration of liver fibrosis (32). Furthermore, LSECs are recognized as important regulators in the progression of liver disease. In pathological conditions of liver fibrosis or cirrhosis, LSECs respond significantly to the stimulation of TNF-α and transforming growth factor (TGF)-β, promoting the expression and secretion of the intermediate factor Midkine (MK) and its receptors, integrins α4 and α6. This process exacerbates LSEC dysfunction through a self-amplifying feedback mechanism, further facilitating the progression of hepatic vascular lesions (33). During intrahepatic sinusoidal remodeling induced by chronic liver injury, LSECs upregulate type IV collagen (COL4) in a TNF-α/NF-κB dependent manner. The deposition of COL4 promotes sinusoidal remodeling and the development of portal hypertension (PHTN) through the activation of angiogenic sprouting of LSECs (34). These findings suggest that restoring the normal function and phenotype of LSECs may represent an effective strategy for treating CLDs.

## Targeting liver sinusoidal endothelial cells

**Figure 1:**
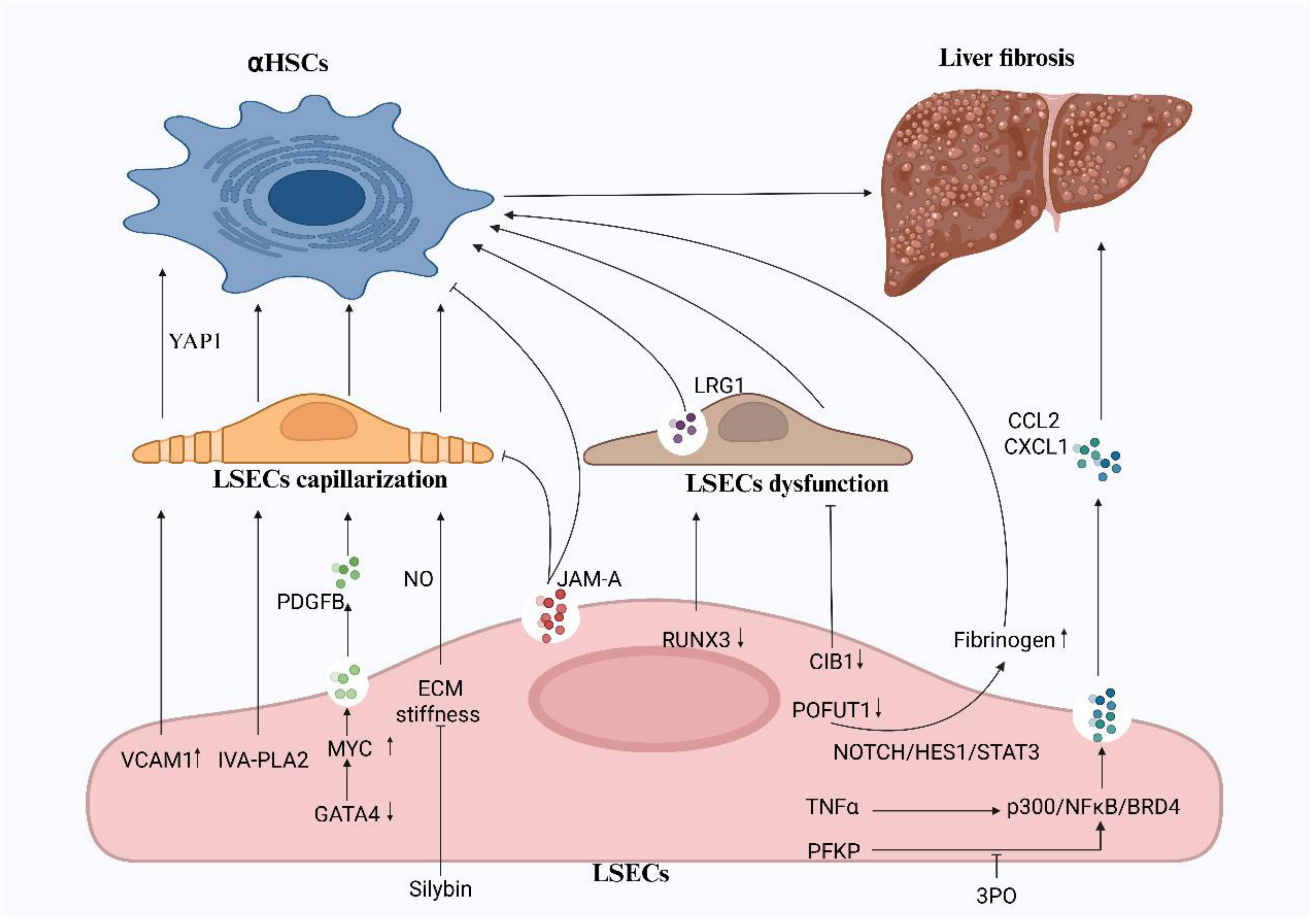
The capillarization and dysfunction of LSECs promote liver fibrosis. The activation of HSCs, driven by the capillarization and functional impairment of LSECs, is a key factor in the progression of liver fibrosis. Targeted inhibition of VCAM1, IVA-PLA2, and ECM stiffness, along with the maintenance of GATA4 expression, can alleviate LSEC capillarization. Silybins have emerged as an effective therapeutic agent in reducing ECM stiffness. while the glycolysis inhibitor 3PO can reduce early-stage liver fibrosis *in vivo*. CIB1, RUNX3, POFUT1, and JAM-A are important factors regulating LSEC function and represent promising targets for the treatment of liver fibrosis. VCAM-1, vascular cell adhesion molecule1; IVA-PLA2, IVA phospholipase A2; CIB1, calcium-and integrin-binding protein1; RUNX3, runt-related transcription factor 3; POFUT1, protein O-fucosyltransferase 1; JAM-A, junctional adhesion molecule A; PFKP, phosphofructokinase 1 isoform P; NF-kB, nuclear factor kappa B; BRD4, bromodomain-containing protein 4. (Created in BioRender).

Given that the transformation and dysfunction of LSECs are pivotal in the onset and progression of liver diseases, we explored the mechanisms of recently identified surface receptors on LSECs, along with other components expressed by these cells, in the context of LSEC-associated liver pathologies (**Figure 1**). Research has shown that LSEC capillarization induced by liver injury mediates the activation of HSCs, a key driver in the development of liver fibrosis (35). In murine models of diet-induced non-alcoholic steatohepatitis (NASH) and liver fibrosis caused by carbon tetrachloride, Guo et al. found that overexpression of LSEC VCAM-1 promotes LSEC capillarization, enhances HSC activation, and accelerates liver fibrosis by activating the Hippo pathway effector YAP1 (36). Furthermore, research has revealed that the expression of endothelial group IVA phospholipase A2 (IVA-PLA2) in LSECs drives sinusoidal capillarization, amplifies HSC activation and contributes to fibrosis, accelerating the progression of NASH. Therefore, developing inhibitors or siRNA-based therapeutics targeting LSEC-specific delivery systems may represent a pivotal strategy for treating NASH-related liver fibrosis (37). The ECM is a crucial component of the LSEC microenvironment, and its impact on LSEC function has drawn significant attention. Studies have demonstrated a mechano transduction mechanism between the ECM and LSECs, revealing that ECM stiffness induces LSEC capillarization via a NO-dependent pathway. Researchers propose that treatment with silybin might alleviate ECM stiffness in fibrotic liver tissue by modulating its biomechanical properties, thereby reducing liver sinusoid capillarization (31). Beyond addressing LSEC capillarization, targeting LSEC-mediated vascular signaling pathways holds potential for advancing liver disease therapies. For instance, TNF-α enhances the expression of CCL2 by promoting the interaction of p300 with NF-kB and BRD4 (38). Greuter et al. have uncovered that mechano transduction-induced glycolysis promotes NF-kB mediated expression of CXCL1, both of which exacerbate liver fibrosis and portal hypertension (39). Consequently, targeting p300 and its binding partners, along with intervention of the glycolytic pathway, may offer promising therapeutic strategies for liver disease. Previous research has identified that the deficiency of the sinusoidal master regulator GATA4 in LSECs drives a profibrotic angiocrine switch and activates HSC by upregulating the expression of the pro-fibrotic vascular secretion factor PDGFB and the transcription factor MYC. These findings highlight the potential therapeutic value of targeting endothelial GATA4 in preventing liver fibrosis and promoting hepatic regeneration (40). A deeper understanding of LSEC function and the underlying mechanisms will be pivotal in the development of novel treatments for liver diseases.

Dysfunctional LSEC is also a key factor involved in the development and progression of liver fibrosis. Numerous factors, such as aging, increased liver stiffness, and abnormal expression of various transcriptional regulators and growth factors, contribute to the functional impairment of LSECs (32, 33, 41, 42). Recently, Wang et al. demonstrated that the expression of CIB1 is significantly increased in LSECs during cirrhosis. Knockdown of CIB1 can improve LSEC function by modulating intracellular tension and inhibiting inflammatory responses (41). Uttam et al. identified RUNX3 as a crucial regulator of the gatekeeping functions of LSECs. Deficiency in RUNX3 leads to LSEC dysfunction and promotes the production of a novel vascular secretion factor, LRG1, which activates HSCs via the TGFBR1-SMAD2/3 signaling pathway. Thus, LRG1 may serve as a potential therapeutic target for liver fibrosis (42). He et al. identified POFUT1 was another critical regulatory factor in LSECs, POFUT1 protects against injury-induced liver fibrosis by inhibiting the expression of fibrinogen (43). Furthermore, previous studies have revealed that the LSEC-specific regulatory factor JAM-A controls the formation of liver sinusoidal capillaries and maintains the quiescent state of HSCs (44). These findings offer valuable insights into the role of LSECs in liver fibrosis and propose potential therapeutic strategies targeting LSEC dysfunction.

In recent years, several drugs targeting LSECs have shown promising therapeutic effects in animal models. Asada and colleagues were the first to demonstrate that tofogliflozin alleviates the progression of perihilar ductal necrosis (PHDN), liver inflammation, and fibrosis by modulating the interplay between LSECs and HSCs in cirrhotic rat models (45). Furthermore, studies have shown that delivering simvastatin specifically to LSECs significantly reduces LSEC capillarization in mouse models. When combined with anti-PD-L1 antibodies, simvastatin nanoparticles showed notable therapeutic efficacy in HCC (46). Mishra and colleagues highlighted that a rationally designed protein ProAgio could induce apoptosis of activated HSCs (aHSCs) and capillarized LSECs, thereby reducing immune cell infiltration and showing potential value in treating advanced NASH and alcoholic hepatitis (AH) (47). In addition, traditional Chinese medicine has shown significant therapeutic effects in HCC progression. Fu et al. demonstrated that Dahuang Zhechong Pill (DHZCP) enhances the capillarization of liver sinusoids in patients with liver cirrhosis and HCC by inhibiting the MK/Itgα signaling pathway of LSECs (48). These studies have paved the way for novel therapeutic approaches to liver fibrosis and related diseases, particularly with a focus on LSECs.

In summary, although current mechanistic studies have identified critical targets that regulate LSEC functions and phenotypes, many therapeutic targets and signaling pathways exhibit multifunctionality across different cell types. Future research should focus on exploring LSEC-specific targets and understanding their interactions with other liver cells within the specialized liver microenvironment, which is crucial for advancing therapeutic strategies for liver fibrosis and related diseases. Additionally, the development of highly targeted nanoparticle-based drug delivery systems, combined with existing immunotherapies, holds the potential to significantly enhance therapeutic efficacy and offer innovative approaches for clinical application.

## Hepatic stellate cells

Residing within the perisinusoidal space of Disse, a specialized microenvironment between sinusoidal endothelial cells and hepatocyte plates, HSCs are specialized resident mesenchymal cells that serve as central mediators of hepatic injury responses through their dual roles in vitamin A storage and fibrogenic activation (49). Under healthy conditions, HSCs remain in a quiescent state with low proliferation rates, primarily has function on the storage and transport of vitamin A, immune modulation, and the support of liver development and regeneration. In the context of chronic liver injury, like that caused by viral infections, ALD and non-alcoholic fatty liver disease (NAFLD), HSCs become activated, undergoing a series of phenotypic transformations. These include transdifferentiation into a myofibroblast-like phenotype, enhanced proliferation and migratory capabilities, and increased chemotaxis. Consequently, activated HSCs produce excessive ECM proteins and release pro-inflammatory mediators, contributing to the progression of liver fibrosis (50, 51). Emerging pathways regulate HSC activation, including autophagy, cellular stress responses, nuclear receptor signaling, energy metabolism, epigenetic modifications and receptor-mediated signaling. Additionally, cytokines and growth factors secreted by resident and infiltrating inflammatory cells within the liver, such as TNF-α, PDGF, and TGF-β1 contribute to HSC activation (49, 52, 53). Activated HSCs play a crucial role in the progression of liver fibrosis by inducing ECM accumulation and secreting various pro-inflammatory cytokines that mediate immune responses, thus perpetuating the inflammatory process (54). Additionally, HSCs promote their own pro-angiogenic phenotype and induce pro-inflammatory phenotypes in other cell types (21, 55). Therefore, strategies targeting the inactivation or apoptosis of activated HSCs hold significant promise for reversing fibrosis. In summary, a comprehensive understanding of the activation mechanisms of HSCs is crucial for developing therapeutic approaches aimed at modulating HSC activity. Progress in this field will provide new insights and strategies for the treatment of liver fibrosis and related diseases in the future.

## Targeting hepatic stellate cells

**Figure 2:**
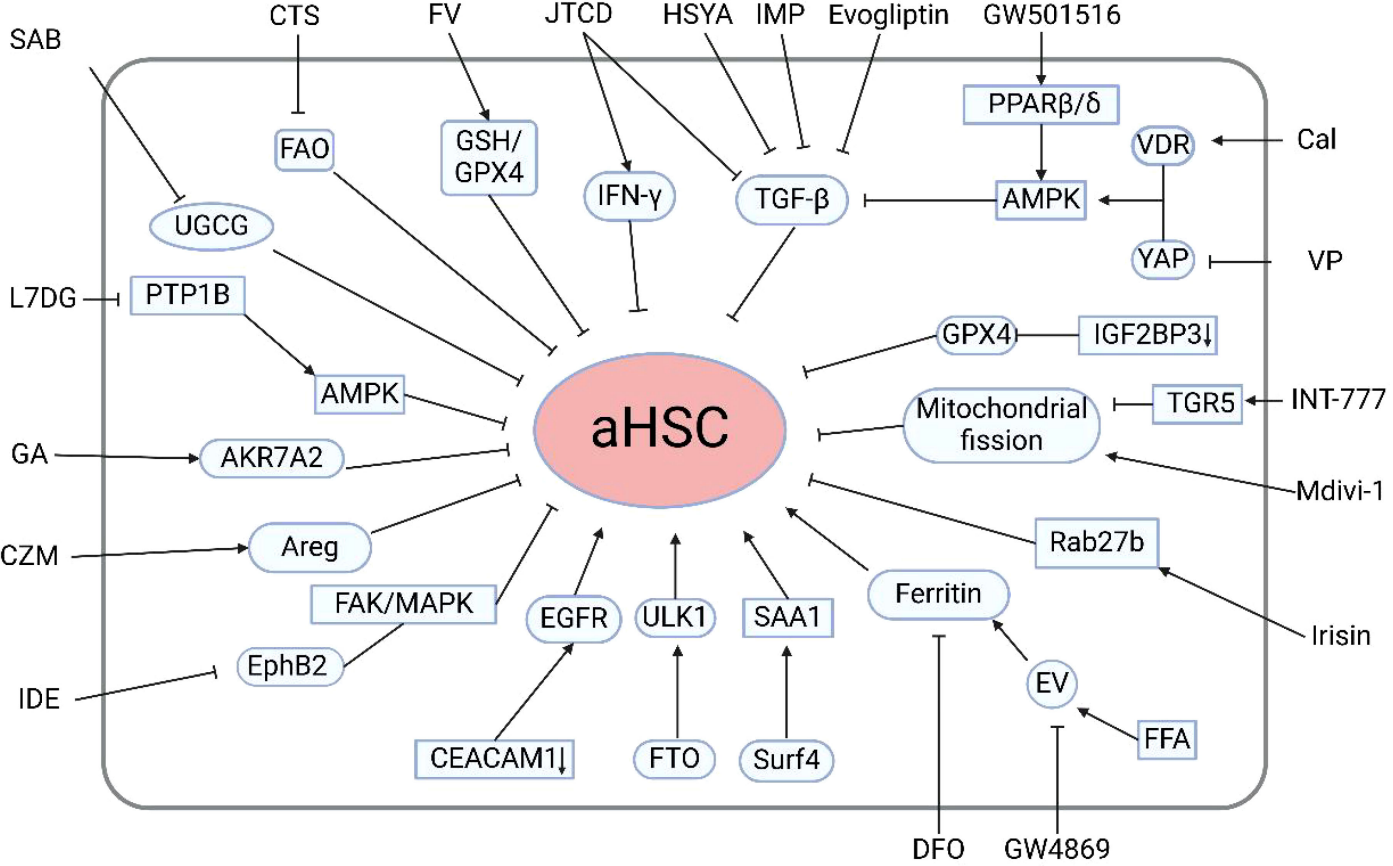
Regulatory mechanisms and therapeutic strategies for modulating HSC activation. Evogliptin, Imperatorin, Jiawei Taohe Chengqi Decoction, hydroxysafflor yellow A, Ficus hirta Vahl, Cryptotanshinone, salvianolic acid B, Luteolin-7-diglucuronide, glycyrrhizic acid, carfilzomib, Idebenone, irisin, INT-777, GW501516, calcipotriol, verteporfin, deferoxamine, and the extracellular vesicle inhibitor GW4869 can suppress hepatic stellate cell activation. Additionally, selective deletion of cell adhesion molecule 1 in HSCs, m6A demethylation of Unc-51-like autophagy activating kinase 1, and surfactant protein 4-mediated secretion of serum amyloid A1 from the liver promote HSC activation. aHSCs, activated HSCs; TGF-β1, transforming growth factor-beta1; FAO fatty, acid oxidation; UGCG, UDP-glucose ceramide glucosyltransferase; PTP1B, protein tyrosine phosphatase 1B; Areg, Amphiregulin; FTO, obesity-associated protein; ULK1, Unc-51-like autophagy activating kinase1; FFA, free fatty acids; PPAR, proliferator-activated receptor; AMPK, AMP-activated protein kinase; VDR, vitamin D receptor; GPX4, glutathione peroxidase 4. (Created in BioRender).

Currently, there are no approved clinical drugs specifically targeting HSCs for the treatment and prevention of early liver disease. However, numerous studies have focused on the mechanisms of HSC activation and proposed corresponding therapeutic strategies (**Figure 2**). Recent studies have highlighted the significant therapeutic potential of traditional Chinese medicine and natural compounds in the treatment of liver diseases. Excessive release of TGF-β1 is one of the critical stimuli inducing HSC activation and their transdifferentiation into myofibroblasts. It has been reported that imperatorin (IMP) significantly inhibits HSC activity by downregulating TGF-β, resulting in reduced liver fibrosis and inflammation. Additionally, IMP has been shown to suppress angiogenesis and vascular remodeling, further contributing to its therapeutic effects (56). Additionally, Ye et al. demonstrated that Jiawei Taohe Chengqi Decoction (JTCD) effectively inhibits HSC activation and reverses liver fibrosis by suppressing the TGF-β1/CUGBP1 signaling pathway and activating the IFN-γ/Smad7 signaling pathway (57). VEGF plays a pivotal role in promoting angiogenesis in the liver. Xue et al. were the first to report the antifibrotic and anti-angiogenic effects of hydroxysafflor yellow A (HSYA), potentially achieved by inhibiting TGF-β1-induced HSC activation and reducing VEGF-A release through the miR-29a-3p/PDGFRB axis (58). Furthermore, studies have shown that Ficus hirta Vahl (FV) can induce HSCs ferroptosis through the glutathione (GSH)/glutathione peroxidase 4 (GPX4) pathway to alleviate liver fibrosis in mouse models (59). Cryptotanshinone (CTS) has been reported to inhibit the activation of mouse HSCs by suppressing STAT3/CPT1A-dependent fatty acid oxidation (FAO) (60). UDP-glucose ceramide glucosyltransferase (UGCG), a key enzyme regulating energy metabolism in HSC activation, has been shown to inhibit HSC activation through inhibiting UGCG by salvianolic acid B (SAB) (61). Another study revealed that protein tyrosine phosphatase (PTP) is involved in the regulation of HSC activation. Luteolin-7-diglucuronide (L7DG) effectively suppresses HSC activation by inhibiting PTP1B activity and activating the phosphorylation of AMP-activated protein kinase (AMPK) (53). Oxidative stress is a crucial factor leading to hepatic oxidative damage. Wang et al. found that glycyrrhizic acid (GA) could reverses the progression of liver fibrosis by inducing the expression of AKR7A2, thus inhibiting ROS-mediated oxidative stress in activated HSCs (62). These studies indicate that herbal medicines and natural compounds regulating HSC activity may represent a novel approach for the treatment of liver diseases.

In addition to natural medicines, multiple studies have reported the potential role of chemical agents in the treatment of liver fibrosis. The proteasome inhibitor carfilzomib (CZM) could be a candidate agent to inhibit HSC activation by reducing the expression of the cytokine amphiregulin (Areg) without causing hepatic injury (63). Idebenone (IDE) significantly inhibits HSC activation. Mechanistically, idebenone inhibits EphB2, a receptor tyrosine kinase associated with liver fibrosis, through its antioxidant activity. The suppression of EphB2 in turn reduces the focal adhesion kinase (FAK)/mitogen-activated protein kinase (MAPK) signaling pathway, further diminishing HSC activation (64). Prior research has indicated that the dipeptidyl peptidase-4 (DPP4) inhibitor evogliptin can markedly suppress the expression of pro-inflammatory cytokines, including IL-1α, IL-1β, TNF-α, IL-6 and TGF-β. These collective actions inhibit HSC activation and mitigate liver fibrosis (65).

Beyond pharmacological treatments, current research is increasingly focused on targeting specific genes, proteins, and associated signaling pathways that mediate HSC activation. Notably, the selective deletion of cell adhesion molecule 1 (CEACAM1) in HSCs induces HSCs activation and promote HSCs transdifferentiation into myofibroblasts through the activation of the EGFR (66). Additionally, autophagy is recognized as a significant contributor to HSC activation and fibrosis. Huang et al. discovered that the obesity-associated protein FTO mediates the m6A demethylation of ULK1, thereby enhancing its expression, increasing autophagy, and ultimately promoting HSC activation (67). Wang et al. recently identified two novel therapeutic targets for liver fibrosis: Surf4 and SAA1. They found Surf4 facilitates the secretion of SAA1 from the liver, thereby activating HSCs and exacerbating fibrosis (68). EVs are critical participants for intercellular communication within the context of NAFLD. Sun et al. demonstrated that treatment with free fatty acids (FFAs) induces EVs and facilitates the release of ferritin from these cells via EVs. Subsequently, the ferritin contained within the EVs is taken up by HSCs, leading to accelerated activation of HSCs. The combination therapy of deferoxamine (DFO) and GW48699, an EV inhibitor, significantly inhibits the progression of NAFLD (69). Conversely, Liao et al. discovered that the cleavage fragment of FNDC5, known as irisin, can inhibit the release of fibrosis-associated EVs and HSC activation by promoting the ubiquitination and degradation of Rab27b (70). Mitochondrial fission has been identified as a key regulatory factor in liver fibrosis. Sun et al. revealed that the TGR5 agonist INT-777 activates TGR5, a bile acid membrane receptor, to suppress mitochondrial fission, thereby inhibiting the activation of HSCs that overexpress hepatitis B virus X protein (71). Additionally, Zhang et al. reported that the PPAR β/δ agonist GW501516 prevents HSC activation and fibrosis. GW501516 acts by activating AMPK, which subsequently inhibits ERK1/2, leading to a reduction in TGF-β1-mediated HSC activation and fibrosis (72). Interestingly, another study reported that the combination of VDR agonist calcipotriol (Cal) and the YAP inhibitor verteporfin (VP) synergistically inhibits HSC activation via AMPK activation (73). Recently, a novel regulatory factor for liver fibrosis, IGF2BP3 has been identified. Specific deletion of IGF2BP3 in HSCs suppresses the expression of GPX4, inducing ferroptosis of HSCs (74). Furthermore, Li et al. designed a prodrug nanoparticle system CREKA-CS-RA (CCR) that targets multiple signaling pathways, effectively delivering inhibitors to the Golgi apparatus of activated HSCs. This system disrupts HSC function and inhibits the Hedgehog signaling pathway, resulting in significant improvement of liver fibrosis (75).

The reversibility of activated HSCs to a quiescent phenotype has emerged as a promising therapeutic strategy for hepatic fibrosis. Emerging evidence demonstrates that multiple pharmacological and molecular interventions can effectively modulate HSC activation dynamics. Yang et al. revealed that astaxanthin exhibits dual therapeutic effects by inhibiting HSC activation and promoting phenotypic reversion of activated HSCs to their quiescent state (76). Miranda et al. demonstrated that triclosan-mediated suppression of fatty acid synthase (FASN) activity activates pro-survival metabolic pathways. This metabolic reprogramming triggers a cascade of cellular and molecular adaptations that facilitate phenotypic reversion of HSCs toward a quiescent-like state (77). Given the pivotal role of TGF-β1 in HSC activation, Cierpka et al. demonstrated that overexpression of PLIN5 facilitates the reversion of activated HSC to a quiescent phenotype by inhibiting the TGF-β1-SMAD2/3 and SNAIL signaling pathways, as well as by reducing the activity of the transcription factor STAT3 (78). Furthermore, Chen et al. developed decellularized ECM scaffolds mimicking the matrix stiffness observed at different fibrosis stages. Their findings indicate that low-stiffness scaffolds effectively promote the reversion of activated HSCs (79).

Future research should focus on elucidating the intricate interplay among HSC-specific genetic regulators, enzymatic networks, and receptor signaling cascades, while simultaneously advancing phenotypic reversal strategies to uncover the molecular mechanisms underlying HSC transdifferentiation and fibrogenesis. This research paradigm must be integrated with the development of multi-target therapeutic platforms for CLDs, particularly given that current HSC-targeted interventions remain largely confined to preclinical models, underscoring the critical need for clinical translation of these experimental agents.

## Kupffer cells

Kupffer cells, the most abundant resident macrophages in the liver, are strategically located within the hepatic sinusoids. These cells exhibit remarkable plasticity, playing dual roles in maintaining liver homeostasis and driving pathological processes (80, 81). Under physiological conditions, KCs act as sentinels of hepatic immunity by phagocytosing pathogens and apoptotic debris, thereby mitigating systemic inflammation. They also secrete anti-inflammatory cytokines such as IL-10 and TGF-β, which promote immune tolerance and support hepatocyte differentiation (82–84). For instance, a recent study revealed that Ca^2+^-activated lipid scramblase TMEM16F (also known as anoctamin 6; ANO6), regulates lipid metabolism to suppress excessive inflammation during Listeria monocytogenes infection (85). Additionally, the erythroblast membrane-associated glycoprotein (ERMAP) on KC surfaces interacts with galectin-9, enhancing their ability to recognize and eliminate cancer cells through lectin-mediated pathways (83).

However, persistent insults like chronic viral infections, alcohol abuse, or lipid overload disrupt this equilibrium. Chronic liver injury promotes the M1 polarization of KCs, characterized by excessive production of TNF-α, IL-6, and IL-1β, alongside ROS and lipid peroxides (86–88). These mediators induce hepatocyte apoptosis and recruit neutrophils and monocytes, amplifying the inflammatory cascade. Furthermore, activated KCs secrete chemokines that promote HSC activation and ECM deposition, directly driving fibrogenesis (89). Pathological inflammation is further exacerbated by gut-derived endotoxins, such as LPS, which bind to TLR4 on KCs, creating a self-perpetuating cycle of injury and fibrosis (90).

Given the multiple roles of KCs in the pathogenesis of chronic liver disease, a deeper investigation into KC-related pro-inflammatory pathways or therapeutic approaches to restore immune homeostasis will become a potential strategy for CLD treatment.

## Targeting Kupffer cells

Acute liver injury and the chronic inflammation are significant contributors to the development of CLDs. The role of KCs in this process is complex: they can alleviate liver damage by releasing anti-inflammatory factors. when excessively activated, they may exacerbate injury by producing pro-inflammatory mediators (91). Therefore, targeting KCs to block their pro-inflammatory activation is of significant importance for the treatment of liver diseases. Recently, numerous publications have reported important regulatory targets for their activity and function (**Table 1**). Research by Zhang and colleagues demonstrated that the deficiency of the inflammation-associated deubiquitinase BRISC (BRCC3 isopeptidase complex) in KCs significantly reduces LPS-induced NF-κB activation, thus decreasing the release of pro-inflammatory cytokines TNF-α, IL-6, and IL-1β. This finding offers novel therapeutic strategies for inhibiting KC-driven inflammation (92). Li et al. reported that silence of the E3 ubiquitin ligase Mid1 resulted in reduced secretion of chemokines CXCL1, CXCL2, CXCL6, and CXCL8, as well as inflammatory factors TNF-α, IL-1β, and IL-6 in KCs by inhibiting the NF-κB, c-JNK, and p38 signaling pathways. This intervention alleviated hepatic ischemia-reperfusion injury (HIRI) (93). Moreover, research by Chao et al. revealed the protective role of KCs against acetaminophen (APAP)-induced acute liver injury, primarily through the inhibition of necroptosis in hepatocytes (94). Additionally, studies using mouse models demonstrated that prolonged exposure to polystyrene (PS) microplastics promotes Kupffer cell pyroptosis. However, knockout of Gsdmd or treatment with the Gsdmd inhibitor necrosulfonamide (NSA) significantly suppressed pyroptosis of KCs, leading to reduced liver inflammatory responses (95).

**Table 1:** Targets of kupffer cells: Their roles and therapeutic strategies in liver diseases.

Targets	Impact on liver diseases	Mechanisms	Treatment strategies	Ref
BRISC	The expression of BRISC in LPS treated KCs promotes the development of ALF	Activates NF-kB, and facilitates the release of pro-inflammatory cytokines	Treatment with thiolutin, a potent BRISC inhibitor	(92)
Mid1	High expression of Mid1 promotes HIRI	Enhances the pro-inflammatory response of KCs and induces neutrophil infiltration	Inhibits Mid1 expression	(93)
Undetermined	KCs alleviate APAP-induced acute liver injury	Suppress hepatocyte necroptosis	Promote the activity of KCs	(94)
GSDMD	GSDMD is associated with acute liver injury induced by PS exposure	Long-term PS exposure promotes KCs pyroptosis and liver inflammation	Knockout of Gsdmd or treatment with the GSDMD inhibitor, NSA	(95)
FASN	Eliminate bacteria in ALD progression	FASN-mediated DNL effectively maintained KCs phagocytosis	Maintaining the expression of FASN	(96)
FFA4	Protect against ethanol-induced hepatic steatosis	N-3 PUFAs activate the FFA4-mediated anti-inflammatory response in KCs	Treatment with FFA4 agonist, compound A (CpdA)	(96)
GPR3	Activation of GPR3 in KCs significantly inhibits the occurrence of NAFLD	GPR3 activation stimulates glycolysis in KCs through GPR3-β-arrestin2-GAPDHPKM2 pathway	Treatment with diphenyleneiodonium (DPI), an agonist of GPR3,	(97)
NCF1	Macrophage NCF1 aggravating MASH progression	NCF1 induces iron deposition and ferroptosis in KCs through OxPLs-TLR4-hepcidin axis	Improve NCF1 expression	(98)
STING	Activation of STING leading to necrotic damage in AIH	Promoting disturbances in hepatic iron ion metabolism as well as oxidative stress response	Inhibit STING expression	(99)
	STING activation contributes to the occurrence and development of RILD	METTL3 promotes the methylation and gene expression of TEAD1, leading to the activation of the STING-NLRP3 signaling pathway	Treatment with H151, a pharmacological inhibitor of STING	(100)
TLR9	Promotes the occurrence and progression of HCC and fibrosis in CLD.	TLR9 is highly expressed in KCs and mediates intercellular signaling during liver injury.	Inhibit TLR9 expression	(101)
TLR8	TLR8 receptor agonist SLGN can effectively inhibit chronic hepatitis B.	Regulate the differentiation status of KCs and impair HBV entry into hepatocytes through an IL-6 dependent mechanism	Treatment with TLR8 agonist, selgantolimod	(102)
LncRNA 220	Alleviate acute liver injury induced by sepsis	Inhibit apoptosis and autophagy in KCs	Increase the expression of LncRNA 220	(103, 104)

In addition to their potential therapeutic roles in acute liver inflammation, the mechanisms by which metabolic reprogramming of KCs contribute to fatty liver disease and alcohol-associated liver disease also warrant further investigation. A recent study indicated that alcohol exposure inhibits FASN-mediated *de novo* lipogenesis (DNL) in KCs, thereby impairing their phagocytic capacity and inducing apoptosis, which promotes the progression of alcoholic liver disease (96). Additionally, Kang et al. identified the free fatty acid receptor 4 (FFA4) as another therapeutic target for alcoholic liver steatosis. Mechanistically, omega-3 polyunsaturated fatty acids (n-3 PUFAs) protect against alcoholic liver steatosis via activating the anti-inflammatory actions of FFA4 on KCs (105). Furthermore, Dong et al. found that activation of the G protein-coupled receptor3 (GPR3) in KCs mediates metabolic reprogramming through the GPR3-β-arrestin2-PKM2 pathway. This finding may provide new avenues for the treatment of NAFLD and obesity (97). Reports also suggest that impaired self-renewal of KCs is a critical factor in the progression of MASH. Zhang et al. found that neutrophil cytosolic factor 1 (NCF1) induces the production of hepcidin in hepatocytes by oxidizing phospholipids, thereby promoting KC iron deposition and MASH (98).

Kupffer cells, as a fundamental component of the liver immune system, exhibit considerable therapeutic potential through their key regulatory factors, which warrant careful consideration. Recent studies have indicated that the stimulator of interferon genes (STING) plays a crucial role in maintaining iron homeostasis in macrophages. Zhao et al. confirmed that activation of STING in KCs promotes dysregulation of iron metabolism, subsequently inducing the development of autoimmune hepatitis (AIH). Ferrostatin-1 (Fer-1) and desferoxamine (DFO) were found to significantly inhibit STING expression (99). Similarly, Wang et al. identified that methyltransferase-like 3 (METTL3) in KCs promotes the methylation and expression of TEAD1, thereby activating the STING-NLRP3 signaling pathway and driving hepatocyte apoptosis. This mechanism offers new therapeutic strategies for radiation-induced liver diseases (RILDs), with STING inhibitors such as H151 demonstrating promising efficacy (100). Furthermore, the Toll-like receptor family serves as important regulators of innate immunity in the liver. Recent studies have revealed elevated expression levels of TLR9 in KCs. The deletion of TLR9 can significantly reduce liver fibrosis by affecting intercellular communication during liver injury (101). Another study demonstrated that the TLR8 agonist selgantolimod (SLGN) can regulate the differentiation status of KCs and impairs HBV entry into hepatocytes via an IL-6-dependent mechanism (102). Interestingly, research conducted by Yang and colleagues has revealed that lncRNA220 acts as a novel modulator of KCs. Mechanistically, lncRNA220 regulates apoptosis and autophagy in LPS-treated KCs through the PI3K-AKT-mTORC1 pathway and the miR-5101/PI3K/AKT/mTOR axis, suggesting that lncRNA220 may serve as a molecular target with clinical, diagnostic, and therapeutic implications in septic acute liver injury (103, 104).

In recent years, the use of nanomaterials as drug delivery carriers in the treatment of various cancers, including HCC, has garnered significant attention (106, 107). Given that KCs are the first line of defense in innate immunity, it is crucial to explore the immune-regulating effects of nanodrugs on KCs and their underlying mechanisms. Recent findings by Jiang et al. elucidated the significant roles of KCs in determining the intrahepatic trafficking of PEGylated liposomal doxorubicin, providing critical insights for further development of nanodrugs (108). Additionally, Ji and colleagues discovered that clodronate-nintedanib-loaded exosome-liposome hybridization enhances the targeted delivery of antifibrotic agents while inhibiting the phagocytic activity of KCs, leading to a reduction in the release of inflammatory cytokines and thereby enhancing the effectiveness of fibrosis treatment (109). Moreover, research by Ding et al. demonstrated that polyethylene glycol-modified graphene oxide (GO-PEG) facilitates the polarization of KCs from M1 to M2, attenuating KC immune activation, which represents an effective strategy for treating liver inflammation (110). Recent studies have also identified that certain natural extracts can regulate the activation and polarization of KCs, influencing the progression of CLDs. For instance, artesunate has been shown to affect M1 macrophage polarization in KCs (111), while gossypetin inhibits their activation (112).

In summary, Kupffer cells, as the first line of defense in the liver immune system, exhibit tremendous potential in the treatment and prevention of liver diseases. Future research should further investigate the specific regulatory mechanisms of KC activity under different liver disease conditions and develop controllable nanoparticle combinations with anti-inflammatory drugs. This approach is anticipated to provide a significant and effective therapeutic strategy for improving treatment outcomes in patients with liver diseases.

## Other innate immune lymphocytes

The liver plays a crucial role in the immune system, serving as a hub for a diverse population of immune cells, including innate immune cells such as NK cells, NKT cells, macrophages, and neutrophils, along with adaptive immune cells like T and B cells. Notably, NK and NKT cells account for approximately one-third of the total lymphocyte population in the liver, and are indispensable for antiviral and anticancer immunity (14, 23). Therefore, exploring the therapeutic potential of NK and NKT cells in the context of liver diseases is of significant importance.

## NK cells

NK cells are a prominent class of non-parenchymal cells (NPCs) within the hepatic immune microenvironment, comprising approximately half of the total lymphocytes in the human liver. These cells play a vital role in maintaining liver immune homeostasis, providing defense against viral infections, and influencing the pathogenesis of various CLDs (113). During HBV infection, NK cells exhibit cytotoxic activity by lysing infected cells and secreting substantial amounts of antiviral cytokines, such as IFN-γ and TNF-α, which contribute to the elimination of tumor cells. Among these cytokines, IFN-γ is recognized as a critical factor for controlling HBV infection (114, 115). It has been reported that HBV infection is closely associated with diabetes-related liver cirrhosis, where the accumulation of S100A8/A9 in patients with cirrhosis and diabetes activates NK cells through receptor for advanced RAGE-mediated p38 MAPK signaling. This activation leads to the secretion of IFN-γ and subsequent necrotic apoptosis of β cells (116). Furthermore, in the liver, the biological activities of different NK cell subsets may have contrasting effects. For instance, persistent high lipid levels reduce UCP1, promoting necrotic apoptosis of NK cells and facilitating the progression of NASH to fibrosis (117). However, recent studies have identified that NK cells are activated in NASH mouse models, secreting a plethora of pro-inflammatory cytokines and chemokines, including IFN-γ, IL-1α, IL-1β, IL-12, GM-CSF, CCL3, CCL4, and CCL5. Simultaneously, they upregulate the JAK-STAT signaling pathway, contributing to hepatocyte injury and the progression of NASH (20). Therefore, specifically regulating the biological activity of NK cells to enhance their cytotoxicity may represent an effective strategy for the treatment of liver diseases.

## NKT cells

NKT cells, a unique subset of T cells that exhibit characteristics of both T cells and NK cells, play significant roles in innate immune responses. Based on the characteristics of their T cell receptors (TCRs), NKT cells can be classified into invariant (iNKT) and non-invariant NKT cell subsets (118). NKT cells respond rapidly to a variety of stimuli and can be effectively activated during immune-mediated liver injury, contributing to the maintenance of immune microenvironment homeostasis and regulating immune functions in a multifaceted way. Specifically, while NKT cells enhance the immune response against tumors and infections, their excessive activation can lead to the release of numerous inflammatory cytokines, triggering a cascade of inflammatory responses and affecting the functions of other immune cells (23, 119). Furthermore, studies indicate that NKT cells play critical roles in the onset and progression of various liver diseases, including ALD, NAFLD, and MAFLD (120–122). Therefore, exploring factors that influence NKT cell function will provide new insights and a solid foundation for designing NKT cell-based immunotherapeutic strategies. This approach is expected to facilitate more precise and personalized immune interventions in the treatment of liver diseases, ultimately improving clinical outcomes for patients.

## Targeting NK cells and NKT cells

In recent years, a range of targeted therapeutic strategies for NK cells has been developed, shedding light on their role in the mechanisms of liver diseases (**Table 2**). Modulating NK cell activity at different stages of liver disease may represent an effective therapeutic approach. For instance, in the context of NASH mediated by NK cell necroptosis induced by high fatty acid levels, Gu et al. found that poly I:C, a TLR3 agonist, effectively reverse cell death and restore UCP1 expression in UCP1^−/−^ NK cells, thereby inhibiting the progression of NASH to fibrosis (117). Wang et al. demonstrated that depleting NK cells, either through NK cell deficiency or the NK cell-neutralizing antibody PK136, alleviates NASH at its early stages (20). Co-infection with HBV and HDV is a significant factor in severe viral hepatitis, and innovative antiviral therapies targeting NK cells have shown notable efficacy. For instance, Groth et al. demonstrated that HDV infection activates NK cells via IFN-β, allowing activated NK cells to eliminate HDV-infected hepatic cell lines via the TRAIL/TRAIL-R2 axis (123). Additionally, co-stimulation of monocytes promotes NK cell release of IFN-γ and TNF-α to combat HBV infection (124). Regulating NK cell-mediated immune homeostasis within the immune microenvironment also presents a viable therapeutic strategy. Guo et al. found that nifedipine increases NK and NKT cell numbers while inhibiting the proliferation of HSCs, impacting liver fibrosis development (129). Immunostimulatory therapies aimed at enhancing the anti-inflammatory cytokine IL-10 and reducing the pro-inflammatory cytokine IL-33 have also been proposed to augment NK cell function (130, 131). Research suggests that reducing the ratio of the inhibitory receptor NKG2A to the activating receptor NKG2D significantly enhances both NK cell immune suppression and anticancer activity (132). Beyond their therapeutic potential, recent studies indicate that the profiling of NK cells may serve as a rapid and valuable tool for assessing the risk of HCC development in hepatitis C virus (HCV) chronically infected patients with cirrhotic liver following HCV cure (133).

**Table 2:** The dual roles of NK and NKT Cells in CLDs and its therapeutic strategies.

Cell Type	Role in CLDs	Key Mechanisms	Therapeutic Targets/Strategies	Ref
NK Cells	NK necroptosis promote NASH progression to fibrosis	Lipid overload induces NK necroptosis by reducing UCP1,	TLR3 agonists (e.g., poly I:C) restore UCP1 expression	(117)
	Antiviral: eliminate HBV/HDV -infected hepatoma cells	Enhances NK activation against HBV/HDV	Enhance NK antiviral activity through IFN-β co-stimulation	(123, 124)
	Pro-inflammatory: NK cell activation promotes NASH progression	Pro-inflammatory cytokine-activated NK cells promote NASH via cytokine-JAK-STAT1/3 axis	Modulate NK cells via PK136 neutralization antibody	(20)
NKT Cells	Antiviral: against hepatitis HBV infection	Restore NKT cell phenotype and antiviral function	Block TREM-1 activation in monocytes and TIGIT	(125, 126)
	Tissue-specific immunometabolic regulation	AMPK expression in iNKT cells ameliorates obesity-induced inflammation	Maintain normal function of the AMPK pathway during obesity-induced inflammation.	(127)
	Antitumor	LCACs inhibit iNKT cell expansion and promote senescence	Reduce LCAC accumulation in tumor tissue.	(128)

Maintaining the activity and function of NKT cells and their subpopulations is considered an ideal strategy for treating liver diseases. For instance, Wu et al. discovered that blocking the activation of triggering receptor expressed on myeloid cells-1 (TREM-1) in monocytes can promote the elimination of HBV by inhibiting pyroptosis in iNKT cells and restoring their functionality (125). Yu et al. found that NKT-like cells express high levels of T-cell immunoreceptor with Ig and ITIM domains (TIGIT), and the blockade of TIGIT can enhance the antiviral capacity of NKT-like cells (126). Excessive activation of the mTORC1/SREBP2 signaling cascade induced by obesity-related NAFLD results in hepatic cholesterol accumulation, which in turn contributes to NKT cell depletion and dysfunction, serving as a key driver for the development of obesity-related HCC. Research indicates that cholesterol-lowering rosuvastatin can restore NKT cell-mediated liver immuno surveillance and prevent obesity-related HCC (134). Moreover, studies have shown that tissue-resident iNKT cells possess unique transcriptional and metabolic characteristics, with AMPK playing a crucial role in ameliorating obesity-induced inflammation in adipose tissue (127). Furthermore, NKT cells are closely linked to inflammation-driven liver damage; recent investigations revealed that T-cell immunoglobulin and mucin domain-containing molecule 4 (Tim-4) serve as an important regulatory factor for maintaining the homeostasis and function of NKT cells under inflammatory stimulation (135). Previous studies suggested that adrenergic signaling can mitigate liver damage caused by iNKT cell activation without affecting their anticancer activity (136). Additionally, Cheng et al. unveiled that the accumulation of long-chain acylcarnitines (LCAC) within tumor tissues can promote the senescence of iNKT cells, and reprogramming LCAC metabolism to restore their anticancer function may represent a novel therapeutic strategy for inflammation-associated tumors (128).

In summary, orchestrating the homeostasis and functions of NK cells and NKT cells may represent a promising strategy for treating liver inflammation and metabolic-related diseases. Future research should delve into the interplay between these cells and the impact of metabolic factors on their functions to uncover the immune mechanisms underlying liver disease, thereby facilitating the development of preventive and therapeutic strategies for metabolic disorders. Additionally, novel immunotherapies targeting NK and NKT cells should be developed to enhance antiviral and anticancer responses.

## Conclusion

CLDs arise from a complex interplay of cellular dysfunction, immune dysregulation, and fibro-inflammatory remodeling, predominantly driven by NPCs. LSECs, HSCs, KCs, and innate lymphoid cells (NK/NKT cells) collectively orchestrate the transition from acute injury to chronic fibrosis and cirrhosis. LSECs initiate sinusoidal dysfunction and HSC activation through capillarization and angiocrine signaling, while HSCs transdifferentiate into collagen-producing myofibroblasts, exacerbating ECM deposition. KCs polarized toward pro-inflammatory phenotypes aggravate hepatocyte injury and promote fibrogenesis via cytokine storms and chemokine secretion. NK/NKT cells exhibit dual roles, balancing antiviral defense with the exacerbation of fibrosis through excessive cytokine production.

Emerging therapeutic strategies targeting NPC-specific pathways, such as modulation of LSEC mechanotransduction, metabolic interventions to inhibit or reverse HSC activation, regulation of KC polarization, and regulation of NK/NKT cell activity. Pharmacological agents (e.g., silybin, fasudil), nanoparticle-based delivery systems, and immunotherapies (e.g., TLR agonists, STING inhibitors) have demonstrated preclinical efficacy in restoring NPC homeostasis. However, challenges persist, including the pleiotropic roles of therapeutic targets across diverse cell populations and the limited clinical translation of preclinical findings. Future research should focus on developing cell-specific interventions, unraveling NPC crosstalk within the hepatic microenvironment, and leveraging multi-omics approaches to refine precision therapies. By aligning mechanistic insights with translational innovation, NPC-targeted strategies offer a promising paradigm to arrest the progression of CLD and mitigate their global burden.
